# A49 AN UNEXPECTED CASE OF SEVERE PROTEIN-LOSING ENTEROPATHY IN A TODDLER

**DOI:** 10.1093/jcag/gwad061.049

**Published:** 2024-02-14

**Authors:** P Podgorny, S Kortbeek, S Lopushinsky, D Boctor

**Affiliations:** University of Calgary, Calgary, AB, Canada; University of Calgary, Calgary, AB, Canada; University of Calgary, Calgary, AB, Canada; University of Calgary, Calgary, AB, Canada

## Abstract

**Background:**

Protein-losing enteropathies (PLE) are characterized by excess loss of serum proteins into the gastrointestinal tract, resulting in hypoproteinemia, peripheral edema, and related respiratory and cardiac sequelae. PLE should be suspected in patients with hypoproteinemia not explained by protein malnutrition, impaired hepatic protein synthesis, or renal losses, and can occur as a consequence of erosive or non-erosive gastrointestinal diseases, and conditions that alter lymphatic flow.

**Aims:**

To present a case of intestinal malrotation with chronic volvulus presenting as PLE.

**Methods:**

In this report, we describe a pediatric patient presenting with PLE ultimately found to have intestinal malrotation with chronic volvulus.

**Results:**

A healthy 1-year-old male presented with acute non-bloody diarrhea and peripheral edema on a background of recurrent colicky abdominal pain and non-bilious emesis. He had significant electrolyte derangements at presentation including hypokalemia, hyponatremia, hypomagnesemia, and metabolic acidosis, with additional laboratory findings of hypoalbuminemia (11g/L), hypogammaglobulinemia (IgA 0.11g/L, IgG 0.77g/L, IgM 0.28g/L), lymphopenia (0.4x10E9/L), and fat-soluble vitamin deficiency (Vitamin A 0.5umol/L, 25-Hydroxyvitamin D 18.4nmol/L, Vitamin E 6umol/L, INR 1.7). In addition to abdominal imaging, he underwent endoscopic evaluation which did not identify an underlying etiology. As his presentation was consistent with PLE, a high-protein, low-fat diet was initiated. Despite dietary adherence for 16 weeks, the PLE features persisted with ongoing severe hypoalbuminemia (20g/L).

Given a suspicion of intestinal lymphangiectasia, a capsule endoscopy was arranged. In the interim, the patient had recurrence of abdominal pain and emesis prompting a repeat abdominal ultrasound which demonstrated swirling of the mesenteric vessels in the midline. An upper gastrointestinal series identified a short mesenteric root with the duodenojejunal junction and cecum in proximity. Urgent laparotomy confirmed a 720-degree volvulus of the small bowel around the mesenteric root, with an abnormally positioned duodenojejunal flexure and cecum. This was presumed to be chronic given the absence of acute vascular compromise. The patient underwent a Ladd procedure. He had complete resolution of symptoms and PLE features on a liberalized diet.

**Conclusions:**

This report identifies a case of intestinal malrotation with chronic volvulus presenting as PLE, highlighting an atypical presentation of intestinal malrotation outside the neonatal period. Awareness of these atypical presentations is important to facilitate prompt diagnosis and treatment, thereby reducing the rate of complications.

**Funding Agencies:**

None

